# A Case of Sporadic Cholera

**Published:** 1890-06

**Authors:** L. Hoopes

**Affiliations:** West Chester, Pa.


### A CASE OF SPORADIC CHOLERA.

L. Hoopes, M. D., West Chester, Pa.

In the summer of 1873, while practicing medicine in Pottstown, Pa., I was summoned hastily one afternoon to see a man residing about four squares from my office, who had been suffering with the following symptoms for about half an hour: About every five minutes he was attacked with cramps from head to foot, drawing him up into a bunch, when he would roll out of bed and around the room like a hoop, making about one circuit of the room, when the cramp would relax and he would vomit a large amount of fluid which looked like rice-water; then he would pass as much more of the same kind of fluid from his bowels, and then, completely exhausted and covered with cold perspiration, the attendants would place him in bed. He was almost pulseless, voice feeble and husky and his skin on being pinched up would remain in a fold; urine suppressed. When I entered the room he was in bed, but I did not ask more than two questions before he had another paroxysm, which I observed closely till it passed, and then dissolved a few pellets of Verat.-alb.[200] in a little water and gave him a teaspoonful before the recurrence of another; in a few minutes there was a slight cramp which ran through the whole body, but not enough to drive him out of bed, a little nausea but no vomiting or stool, nor did they recur again. I gave him no more Verat., but on the following day I gave him a dose or two of Chin.[200] for the remaining debility, and he made a good and rapid recovery. In the paroxysm which I witnessed the fluid passed by mouth and anus filled a large chamber.

There were three of these cases, almost exactly alike, which occurred on three consecutive days. The first recovered under eclectic treatment, and the third died under allopathic.

